# Wrapper-based selection of genetic features in genome-wide association studies through fast matrix operations

**DOI:** 10.1186/1748-7188-7-11

**Published:** 2012-05-02

**Authors:** Tapio Pahikkala, Sebastian Okser, Antti Airola, Tapio Salakoski, Tero Aittokallio

**Affiliations:** 1 Department of Information Technology, University of Turku, Turku, Finland; 2 Turku Centre for Computer Science, Turku, Finland; 3 Department of Mathematics, University of Turku, Turku, Finland; 4 Data Mining and Modeling group, Turku Centre for Biotechnology, Turku, Finland; 5 Institute for Molecular Medicine Finland (FIMM), University of Helsinki, Helsinki, Finland

**Keywords:** GWAS, Genome-wide association study, Machine learning, Feature selection

## Abstract

**Background:**

Through the wealth of information contained within them, genome-wide association studies (GWAS) have the potential to provide researchers with a systematic means of associating genetic variants with a wide variety of disease phenotypes. Due to the limitations of approaches that have analyzed single variants one at a time, it has been proposed that the genetic basis of these disorders could be determined through detailed analysis of the genetic variants themselves and in conjunction with one another. The construction of models that account for these subsets of variants requires methodologies that generate predictions based on the total risk of a particular group of polymorphisms. However, due to the excessive number of variants, constructing these types of models has so far been computationally infeasible.

**Results:**

We have implemented an algorithm, known as greedy RLS, that we use to perform the first known wrapper-based feature selection on the genome-wide level. The running time of greedy RLS grows linearly in the number of training examples, the number of features in the original data set, and the number of selected features. This speed is achieved through computational short-cuts based on matrix calculus. Since the memory consumption in present-day computers can form an even tighter bottleneck than running time, we also developed a space efficient variation of greedy RLS which trades running time for memory. These approaches are then compared to traditional wrapper-based feature selection implementations based on support vector machines (SVM) to reveal the relative speed-up and to assess the feasibility of the new algorithm. As a proof of concept, we apply greedy RLS to the Hypertension – UK National Blood Service WTCCC dataset and select the most predictive variants using 3-fold external cross-validation in less than 26 minutes on a high-end desktop. On this dataset, we also show that greedy RLS has a better classification performance on independent test data than a classifier trained using features selected by a statistical p-value-based filter, which is currently the most popular approach for constructing predictive models in GWAS.

**Conclusions:**

Greedy RLS is the first known implementation of a machine learning based method with the capability to conduct a wrapper-based feature selection on an entire GWAS containing several thousand examples and over 400,000 variants. In our experiments, greedy RLS selected a highly predictive subset of genetic variants in a fraction of the time spent by wrapper-based selection methods used together with SVM classifiers. The proposed algorithms are freely available as part of the RLScore software library at http://users.utu.fi/aatapa/RLScore/.

## Background

The common goal of genome-wide association studies (GWAS) is the identification of genetic loci that can help to discriminate an individual’s susceptibility to various common disorders. Identification of genetic features that are highly predictive of an individual’s disease status would facilitate the development of methods for determining both an individual’s risk of developing a clinical condition along with the possibility of new treatment options such as personalized medicine [1-5]. In the case of GWAS, the common genetic marker of interest is the single nucleotide polymorphism (SNP). It is widely theorized that complex diseases can be predicted before an individual has been found to have a clinical manifestation of a particular disorder [4,6,7]. The creation of more accurate disease risk detection techniques will ideally assist clinicians in the development of new medicines in addition to determining which individuals are in a greater need of receiving expensive preventative treatments, while allowing those who are at a low risk to avoid undergoing potentially superfluous medical care.

While numerous genetic loci have been prior identified through standard SNP analyses, the results of these studies have only provided a limited explanation regarding an individual’s disease status [3,5,7-9]. Contrary to the current knowledge of synergistic interactions amongst genetic variants, traditional GWAS, through the use of single-SNP association testing, have implemented analysis methodologies that ignore the epistasis interactions between the genetic loci [3,7,10-12]. While it has been prior demonstrated that the heritability of most disorders is the result of numerous complex interactions between multiple SNPs, the aggregate of the effects of these markers still provides a clinically insufficient prediction of the disease status [10,13]. To account for these variant interactions, association studies have begun to implement various machine learning-based approaches to incorporate the complex epistasis pattern effects [3,7,14-16]. In contrast to conventional statistical methods, machine learning algorithms tend to place a larger emphasis on prediction making and how the values of a particular variant contribute to the effect of other markers, making them ideal for developing predictive strategies in genetic association studies.

In typical GWAS, the problems under study are modeled as binary classification tasks. Examples are labeled either as cases or controls for a particular disease, with the cases representing those individuals who have the disease and the controls those who are free of the disease. In recent years, methods of selecting the most relevant variants to prediction of a disease, known as feature selection, have begun to gain prominence in bioinformatics studies [7,17-20]. Two common feature selection methodologies are commonly presented, filter and wrapper methods [17,18]. In filter methods, the selection is done as a pre-processing step before learning by computing univariate statistics on feature-by-feature basis. The approach is computationally efficient, but the methods are not able to take into account the dependencies between the variants, or the properties of the learning algorithm which is subsequently trained on the features. This can lead to suboptimal predictive performance.

Delving deeper into feature selection, we consider the wrapper model, in which the features are selected through interaction with a classifier training method [21]. The selection consists of a search over the power set of features. For each examined feature set, a classifier is trained, and some scoring measure, which estimates its generalization error, is used to evaluate the quality of the considered feature set. Measuring the feature set quality on the training set is known to have a high risk of overfitting, and hence other estimates, such as those based on cross-validation (CV) [22,23], have been proposed as more reliable alternatives (see e.g. [21]). Since the size of the search space grows exponentially with the number of features, testing all feature subsets is infeasible. Rather, wrapper methods typically use search heuristics, such as greedy forward or backward selection, or genetic algorithms, to find locally optimal solutions. The wrapper methods have been demonstrated to have the potential to achieve better predictive performance than the filter approach [7,18,24,25], but this increase in performance is accompanied by increased computation times. This is due to the property of the wrapper methods that they require re-training a classification algorithm for each search step and each round of CV.

A number of studies related to the use of wrapper-based feature selection and the implementation of classifiers on biological markers haven been published, with the majority of the work dealing with the problem of gene selection from DNA microarray data. One of the most successful classifier learning algorithms in this domain has been the support vector machine (SVM) [26]. Proposed approaches include the combination of SVM classification with pre-filtering of features [27,28], wrapper based methods [29-31], as well as embedded methods that incorporate feature selection within the SVM training algorithm, such as the recursive feature selection method [32]. These previous approaches have been mostly proposed for and tested on small scale learning problems, where the number of training examples ranges in at most hundreds, and the number of features in thousands. However, it is not straightforward to extend these methods to GWAS problems, where the training set sizes range in thousands and feature set sizes in hundreds of thousands or even millions. From a scalability perspective, SVMs are actually not a particularly suitable choice as a building block for constructing feature selection methods, since the method has to be re-trained from scratch for each tested feature set, and for each round of CV. This can lead to unfeasible computational costs on large and high-dimensional data sets. Due to this reason, previous studies on implementing SVMs on GWAS have required pre-filtering of the data [3,20,33]. The same problem naturally applies also to most other classifier training methods.

Regularized least-squares (RLS), also known as the least-squares support vector machine (LS-SVM), and ridge regression, among other names, is a learning algorithm similar to SVMs [34-40]. Numerous comparisons of the SVM and RLS classifier can be found in the literature (see e.g. [37,40-43]), the results showing that typically there is little to no difference in classification performance between the two methods. However, for the purposes of wrapper based feature selection, RLS has one major advantage over SVMs, namely that RLS has a closed form solution that can be expressed in terms of matrix operations. This in turn allows the development of computational shortcuts, which allow re-using the results of previous computations when making minor changes to the learning problem. The existence of a fast leave-one-out (LOO) CV shortcut is a classical result [44], that has recently been extended to arbitrarily sized folds [45]. Similar shortcuts can be developed for operations where features are added to the, or left out of the training set, and the resulting classification model is updated accordingly. Such shortcuts have been used to derive RLS-based wrapper selection methods for gene selection from microarray data [19,46-48]. However, the previously proposed methods did not fully utilize the possibilities of matrix algebra for speeding up the computations, making them still unsuitable for very large data sets, such as those encountered in GWAS.

In the present work, we have developed and implemented the first wrapper-based feature selection method capable of performing feature selection on the entire span of SNPs available in a typical GWAS, without the necessity for pre-filtering to reduce the number of attributes. The method is based on the greedy RLS algorithm [49], which uses computational shortcuts to speed up greedy forward selection with LOO error as the selection criterion. Greedy RLS yields equivalent results to the most efficient of the previously proposed methods for wrapper based feature selection with RLS, called the low-rank updated LS-SVM method [48], while having lower computational complexity. Namely, the running time of greedy RLS grows linearly in the number of training examples, the number of features in the original data set, and the number of selected features. This is in contrast to the low-rank updated LS-SVM that scales quadratically with respect to the number of training data points. Further, we propose a space-efficient variation of greedy RLS that trades speed for decreased memory consumption. The method is efficient enough to perform feature selection on GWAS data with hundreds of thousands of SNPs and thousands of data points on a high-end desktop machine. As a case study, we were able to implement the method on the Wellcome Trust Case-Control Consortium (WTCCC) Hypertension (HT) dataset combined with the National Blood Service (NBS) controls samples, obtaining a highly discriminant classification on independent test data.

### Related works

There exists a number of prior works in applying machine learning based method to GWAS studies. For instance, it was demonstrated that when SVMs are applied to the results of filter based feature selection, high area under the curve (AUC) values in the detection of Type 1 Diabetes (T1D) can be obtained [3]. More specifically, it was shown that through the use of a filter method, in which they selected only those features with significance values of less than pre-selected thresholds, they could outperform logistic regression methods. The paper made the discerning observation that using only more statistically significant markers in disease prediction actually causes a loss of information and thus a decrease in AUC [3]. Such statistical p-value based filtering has also been shown to result in sub-optimal prediction performance in other studies [2,50,51].

Previously, we have shown that in a population based candidate SNP study, a combined filter-wrapper approach allowed for an accurate prediction of the onset of carotid atherosclerosis on independent test data [7]. While the accuracy of the wrapper-based methods was demonstrated on a small subsample of available SNPs, the method would not scale to unfiltered SNP sets. Also other approaches, such as dimensionality reduction, have been applied, but they were not able to scale to an entire GWAS either [52]. Moreover, LASSO-based feature selection methods have been used, but only on a filtered-subset rather than an entire GWAS [12,53]. Furthermore, several other works have also addressed the issue of the computational feasibility of implementing machine learning algorithms on entire GWAS but have reported the same conclusion, that at the moment it was not practical to use such methods without extensive pre-filtering [15,54-56].

To conclude, the above mentioned works tend to make use of various filters to initially reduce the total number of features to a number in which computationally non-optimized algorithms can be applied. Most works tend to filter the final number of SNPs being analyzed to the tens of thousands. While such methods are often sufficient for analyzing GWAS datasets, our aim here is to show that it is computationally feasible to implement wrapper methods on entire GWAS scale with a large number of training examples and all of the available features. This, in turn, can lead to discovering models with increased predictive performance, as is shown in our experiments.

## Methods

### Preliminaries

Let us start by making the assumption that the task being solved is a binary classification problem. We are supplied with a training set of *m* examples, each having *n* real-valued features, as well as a class label denoting whether the example belongs to the positive or to the negative class. In the case of GWAS, the features are representative of the number of minor alleles present in a particular SNP (either 0, 1 or 2, representing the minor allele count), the examples represent each individuals data in a particular study and the class label is the disease status of a particular example, with the positive class representing those who have the disease and the negative class indicative of those without the disease. Our goal is to select an informative subset of the features, based on which we can construct an accurate classifier for predicting the class labels of new, unseen test examples.

Next, we introduce some matrix notation. Let

$$R^{m}$$

and

$$R^{n\times m}$$

denote the sets of real-valued column vectors and *n*×*m*-matrices, respectively. To denote real valued matrices and vectors we use bold capital letters and bold lower case letters, respectively. Moreover, index sets are denoted with calligraphic capital letters. By denoting **M**_*i*_, **M**_:,*j*_, and **M**_*i*,*j*_, we refer to the *i*th row, *j*th column, and (*i*,*j*)th entry of the matrix

$$M\in R^{n\times m}$$,

respectively. Similarly, for index sets

R⊆{1,…,n}

and

L⊆{1,…,m},

we denote the submatrices of **M** having their rows indexed by

R,

the columns by

L,

and the rows by

R

and columns by

L

as

$$M_{R}$$,

$$M_{:,L}$$,

and

$$M_{R,L}$$,

respectively. We use an analogous notation also for column vectors, that is, **v**_*i*_ refers to the *i*th entry of the vector **v**.

Let

$$X\in R^{n\times m}$$

be a matrix containing the whole feature representation of the examples in the training set, where *n* is the total number of features and *m* is the number of training examples. The (*i*,*j*)th entry of **X**contains the value of the *i*th feature in the *j*th training example. Note that while we here define **X** to be real-valued, in GWAS the data can usually be stored in an integer-valued matrix, which is much more memory efficient. The memory issues concerning the data types are discussed more in detail below. Moreover, let

$$y\in R^{m}$$

be a vector containing the labels of the training examples. In binary classification tasks, we restrict the labels to be either 1 or −1, indicating whether the data point belongs to the positive or negative class, respectively.

In this paper, we consider linear predictors of type

(1) $$f(x)=w^{T}x_{S},$$

where **w** is the

|S|

-dimensional vector representation of the learned predictor and

$$X_{S}$$

can be considered as a mapping of the data point *x* into

|S|

-dimensional feature space.^a^ Note that the vector **w** only contains entries corresponding to the features indexed by

S.

The rest of the features of the data points are not used in the prediction phase. The computational complexity of making predictions with (1) and the space complexity of the predictor are both

O(|S|)

provided that the feature vector representation

$$X_{S}$$

for the data point *x* is given.

### Wrapper-based feature selection

In wrapper-based feature selection, the most commonly used search heuristic is greedy forward selection in which one feature is added at a time to the set of selected features, but features are never removed from the set. A pseudo code of a greedy forward selection that searches feature sets up to size *k*, is presented in Algorithm 1. In the algorithm description, the outermost loop adds one feature at a time into the set of selected features

S

until the size of the set has reached the desired number of selected features *k*. The inner loop goes through every feature that has not yet been added into the set of selected features and, for each of those, computes the value of the heuristic H for the set including the feature under consideration and the current set of selected features. With

$$\text{H}(X_{R},y)$$,

we denote the value of the heuristic obtained with a data matrix

$$X_{R}$$

and a label vector **y**. In the end of the algorithm description,

$$t(X_{S},y)$$

denotes the black-box training procedure which takes a data matrix and a label vector as input and returns a vector representation of the learned predictor **w**.

### Algorithm 1 Wrapper-based feature selection

Using the training set error as a selection heuristic is known to be unreliable due to overfitting, and therefore it has been proposed to measure the quality of feature sets with CV [57]. The CV approach can be formalized as follows. Let

C={1,…,m}

denote the indices of the training instances. In CV, we have a set

$$H=\{H_{1},\ldots ,H_{N}\}$$

of hold-out sets, where

N∈N

is the number of rounds in CV and

$$H_{i}\subseteq C$$.

In the most popular form of *N*-fold CV, the hold-out sets are mutually disjoint, that is,

$$H_{i}\cap H_{j}=\emptyset$$

when *i*≠*j*. Now, given a performance measure *l*, the average performance over the CV rounds is computed as

$$L(X,y)=\underset{H\in H}{\sum }l\left(f_{\bar{H}}(X_{:,H}),y_{H}\right),$$

where

$$f_{\bar{H}}$$

is a predictor which is trained with the examples indexed by

$$\bar{H}=C\setminus H$$,

and

$$X_{:,H}$$

and

$$y_{H}$$

contain, respectively, the features and the labels of the examples indexed by

H.

Leave-one-out (LOO) CV is an extreme form of *N*-fold CV in which every hold-out set is of size one and every training example is held out at a time, that is, *N*=*m*.

Since the outer and inner loops in Algorithm 1 have *k* and *n* rounds, respectively, the computational complexity of the wrapper based greedy forward selection is *O*(*knH*), where *H* is the complexity of calculating the value of the heuristic for feature sets of size up to *k*. For example, if we use LOO error as a heuristic and the LOO calculation is wrapped around a black-box training algorithm, the time complexity of the heuristic is usually *m* times the complexity of the training method. This is often infeasible in practice. Fortunately, as it is widely known in literature, computational short-cuts enabling the calculation of the LOO error without needing to retrain the predictor from scratch exist for many machine learning methods (see e.g. [23]).

The selection of the performance measure *l* used in the CV heuristics may also have an effect on the computation time. The performance measure can be selected to be the same as the one we aim to maximize in the first place but it may also make sense to use approximations in order to speed up the feature selection process. For example, while the computation of AUC requires *O*(*m*log(*m*))floating point operations, the mean squared error can be computed in a linear time. These complexities are, of course, usually negligible compared to the training complexities of the learning methods. However, this is not the case for the greedy RLS method as we will show below.

### Support vector machines and regularized least squares

A large class of machine learning algorithms can be formulated as the following regularized risk minimization problem [58]:

(2) $$w^{*}=\underset{w\in R^{\left|S\right|}}{\text{argmin}}\left\{l\left({\left(X_{S}\right)}^{T}w,y\right)+\lambda w^{T}w\right\},$$

where the first term is the empirical risk measuring how well **w** fits the training data, **w**^T^**w**is the quadratic regularizer measuring the complexity of the considered hypothesis, *λ*>0 is a parameter, and

$$l:R^{m}\times R^{m}\mapsto [0,\infty )$$

is a convex loss function measuring how well a predicted and true label match. The regularized risk minimization framework (2) can be extended to non-linear learning and structured data by means of the *kernel trick*[59], however this is not necessary for the considerations in this paper.

*The hinge loss*, defined as

(3) $$l\left({\left(X_{S}\right)}^{T}w,y\right)=\overset{m}{\underset{i=1}{\sum }}\max\left(1-y_{i}{\left({\left(X_{S}\right)}^{T}w\right)}_{i},0\right),$$

leads to the soft margin Support Vector Machine (SVM) problem^b^[26], when inserted into equation (2).

*The squared loss*, defined as

(4) $$l\left({\left(X_{S}\right)}^{T}w,y\right)={\left({\left(X_{S}\right)}^{T}w-y\right)}^{T}\left({\left(X_{S}\right)}^{T}w-y\right),$$

leads to the Regularized Least-Squares (RLS) problem [34-40].

### Greedy regularized least-squares

We next recall the description of greedy RLS, our linear time algorithm for greedy forward selection for RLS with LOO criterion, which was introduced by us in [49]. A detailed pseudo code of greedy RLS is presented in Algorithm 2.

### Algorithm 2 Greedy RLS

First, we consider finding a solution for the regularization problem (2) with the squared loss (4) for a fixed set of features

S:

(5) $$\underset{w\in R^{\left|S\right|}}{\text{argmin}}\left\{{\left({\left(X_{S}\right)}^{T}w-y\right)}^{T}\left({\left(X_{S}\right)}^{T}w-y\right)+\lambda w^{T}w\right\}.$$

By setting the derivative of (5) with respect to **w** to zero, we get

(6) $$\begin{array}{lcrr} w & = & {\left(X_{S}{\left(X_{S}\right)}^{T}+\lambda I\right)}^{-1}X_{S}y & \end{array}$$

(7) $$\begin{array}{lcrr} & = & X_{S}{\left({\left(X_{S}\right)}^{T}X_{S}+\lambda I\right)}^{-1}y, & \end{array}$$

where **I** is the identity matrix and the second equality is due to the well-known matrix inversion identities (see e.g. [60]).

Before continuing, we introduce some extra notation. Let

(8) $$G={\left({\left(X_{S}\right)}^{T}X_{S}+\lambda I\right)}^{-1}.$$

While the matrix **G**is only implicitly used by the algorithms we present below, it is nevertheless a central concept in the following considerations. Moreover, let

(9) $$\begin{array}{lll} a & =Gy,\; & \;\\ d & =\text{diag}(G),\; & \;\\ C & =GX^{T},\; \end{array}$$

where diag(**G**) denotes a vector that consist of the diagonal entries of **G**. In the literature, the entries of the vector

$$a\in R^{m}$$

are often called the dual variables, because the solutions of (5) can be equivalently expressed as

$$w=X_{S}a$$,

as can be observed from (7).

Next, we consider a well-known efficient approach for evaluating the LOO performance of a trained RLS predictor (see e.g. [23,61]). Provided that we have the vectors **a** and **d**available, the LOO prediction for the *j*th training example can be obtained in constant number of floating point operations from

(10) $$y_{j}-{\left(d_{j}\right)}^{-1}a_{j}.$$

We note that (10) can be further generalized to hold-out sets larger than one (see e.g. [45]).

In order to take advantage of the computational short-cuts, greedy RLS maintains the current set of selected features

S⊆{1,…,n},

the vectors

$$a,d\in R^{m}$$

and the matrix

$$C\in R^{m\times n}$$.

In the initialization phase of the greedy RLS algorithm (lines 1-4 in Algorithm 2) the set of selected features is empty, and hence the values of **a**, **d**, and **C** are initialized to *λ*^−1^**y**, *λ*^−1^**1**, and *λ*^−1^**X**^T^, respectively, where

$$1\in R^{m}$$

is a vector having every entry equal to 1.

The middle loop of Algorithm 2 traverses through the set of

n−|S|

available features and selects the one whose addition decreases the LOO error the most. The innermost loop computes the LOO error for RLS trained with features

S∪{i}

with formula (10). For this purpose, the vectors **a**and **d** must be modified so that the effect of the *i*th feature is removed. In addition, when the best feature is found, it is permanently added into

S

after which the vectors **a** and **d** as well as the matrix **C** are updated. Since the definitions of **a**, **d**, and **C** all involve the matrix **G**, we first consider how the feature additions affect it. We observe that **G** corresponding to the feature set

S∪{i}

can be written as

(11) $$\begin{array}{ll} \tilde{G} & ={\left({\left(X_{S}\right)}^{T}X_{S}+{\left(X_{i}\right)}^{T}X_{i}+\lambda I\right)}^{-1}\; \end{array}$$

(12) $$\begin{array}{ll} & =G-uX_{i}G,\; \end{array}$$

where

(13) $$u=C_{:,i}{\left(1+X_{i}C_{:,i}\right)}^{-1}.$$

The equality (12) is due to the well-known Sherman-Morrison-Woodbury formula (see e.g. [60]). Accordingly, the vector

$$\tilde{a}$$

corresponding to

S∪{i}

can be written as

(14) $$\begin{array}{lll} \tilde{a} & =(G-uX_{i}G)y\; & \;\\ & =a-u(X_{i}a),\; \end{array}$$

the *j*th entry of

$$\tilde{d}$$

as

(15) $$\begin{array}{lll} {\tilde{d}}_{j} & ={\left(G-uX_{i}G\right)}_{j,j}\; & \;\\ & ={\left(G-u{\left(C_{:,i}\right)}^{T}\right)}_{j,j}\; & \;\\ & =d_{j}-u_{j}C_{j,i},\; \end{array}$$

and the cache matrix **C**as

$$C-u(X_{i}C).$$

By going through the matrix operations in the pseudo code of greedy RLS in Algorithm 2, it is easy to verify that the computational complexity of the whole algorithm is *O*(*kmn*), that is, the complexity is linear in the number of examples, features, and selected features. Considering this in the context of the analysis of wrapper-based feature selection presented above, this means that the time spent for the selection heuristic is *O*(*m*), which is far better than the approaches in which a black-box training algorithm is retrained from scratch each time a new feature is selected.

### Space efficient variation

The computational efficiency of greedy RLS is sufficient to allow its use on large scale data sets such as those occurring in GWAS. However, the memory consumption may become a bottleneck, because greedy RLS keeps the matrices

$$X\in R^{n\times m}$$

and

$$C\in R^{m\times n}$$

constantly in memory. In GWAS, the data matrix **X**usually contains only integer-valued entries, and one byte per entry is sufficient for storage. In contrast, the matrix **C** consists of real numbers which are in most systems stored with at least four bytes per entry.

In this section, we present a variation of greedy RLS which spends less memory when dealing with large data sets. Namely, the proposed variation avoids storing the cache matrix **C**in memory, and hence the memory consumption is dominated by storing the matrix **X**. The savings can be significant if the training data is integer valued, such as in SNP datasets.

The pseudo code of this variation is given in AlgorithmAlgorithm 3 Space Efficient Greedy RLS. Next, we describe its main differences with Algorithm 2 and analyze its computational complexity and memory consumption in detail. Formally, let

r=min(m,|S|)

and let

$$X_{S}=U\Sigma V^{T}$$

be the economy-size (see e.g. [62]) singular value decomposition (SVD) of

$$X_{S}$$,

where

$$U\in R^{|S|\times r}$$

and

$$V\in R^{m\times r}$$

contain the left and the right singular vectors of **X**, respectively, and

$$\Sigma \in R^{r\times r}$$

is a diagonal matrix containing the corresponding singular values. Note that

$$X_{S}$$

has at most *r* nonzero singular values. Since we use the economy-size SVD, where we only need to store those singular vectors that correspond to the nonzero singular values, the size of the matrices **U** and **v**is determined by *r*. The computational complexity of the economy-size SVD of

$$X_{S}$$

is

$$O(\min(m^{2}|S|,m|S{|}^{2}))$$

(see e.g. [62]). Substituting the decomposed data matrix into (8), we get

$$\begin{array}{lll} G & ={\left(X^{T}X+\lambda I\right)}^{-1}\; & \;\\ & ={\left(V\Sigma^{T}U^{T}U\Sigma V^{T}+\lambda I\right)}^{-1}\; & \;\\ & ={\left(V\Sigma^{T}\Sigma V^{T}+\lambda I\right)}^{-1}\; & \;\\ & =V({\left(\Sigma^{T}\Sigma +\lambda I\right)}^{-1}-\lambda^{-1}I)V^{T}+\lambda^{-1}I\; & \;\\ & =V\Omega V^{T}+\lambda^{-1}I,\; & \; \end{array}$$

where

$$\Omega ={\left(\Sigma^{T}\Sigma +\lambda I\right)}^{-1}-\lambda^{-1}I$$

and the dimensions of the identity matrices are either *r*×*r*or *m*×*m* depending on the context. Note that inverting ***Σ***^T^***Σ*** + *λ***I**requires only *O*(*r*)time, because it is a diagonal matrix. Now, the *i*th column of the matrix **C** can be written as

(16) $$c=V(\Omega (V^{T}{\left(X_{i}\right)}^{T}))+\lambda^{-1}{\left(X_{i}\right)}^{T}$$

which can be computed in *O*(*mr*)time.

### Algorithm 3 Space Efficient Greedy RLS

**Figure 1:**
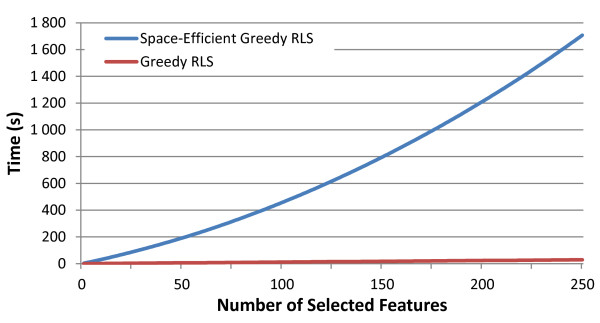
**Comparison of the greedy RLS implementations.** Plot showing the timing comparison (in seconds) for the two variations of greedy RLS. Note the linearity in the greedy RLS curve compared to the quadratic nature of the space-efficient version with respect to the number of selected features. The run was based on randomly sampled datasets with 1,000 training examples and 10,000 features.

If *k* is the number of features that will be selected, SVD has to be computed *k* times, resulting in complexity *O*(min(*k*^3^*m*,*k*^2^*m*^2^)). The computation of (16) is performed *O*(*kn*) times resulting in a complexity *O*(min(*k*^2^*mn*,*km*^2^*n*)), which dominates the overall computational complexity of this variation. Since storing and updating the cache matrix **C** is not required in Algorithm 3, the memory consumption is dominated by the data matrix **X**, which can, in the context of GWAS data, be stored as an array of integers. In addition, computing and storing the right singular vectors requires a real valued matrix of size *m*×*r*. However, this has a negligible memory consumption unless both *k* and *m* are close to *n*, which is usually not the case in GWAS. To conclude, in GWAS experiments, the memory consumption of Algorithm 3 is about one fifth of that of Algorithm 2 because it avoids storing **C** that requires four bytes of memory per entry whereas **X**requires only one. The timing comparison of the space efficient model when compared with the normal greedy RLS can be seen in Figure 1.

## Results and discussion

In the experiments, we first demonstrate the scalability of the greedy RLS method to large-scale GWAS learning. As a point of comparison, we present runtimes for a wrapper-based selection for an SVM classifier to which we refer as SVM-wrapper. The greedy RLS algorithm was implemented in C++ to allow for minimal overhead with regards to looping over large datasets and to allow efficient future adaptations of the code, such as parallelization to take advantage of both shared and distributed memory systems. The space-efficient version of the greedy RLS method was implemented in Python, in order to make use of its well established numerical analysis packages for computing the required singular value decompositions. For the SVM-wrapper, we chose to use the LibSVM in Weka 3.7.3 [63,64] and LibSVM in the e1071 package in R [65-67]. This choice was made because these environments have been commonly used in other studies that have attempted to solve similar problems, and since the LibSVM package itself is known to be one of the most efficient existing SVM implementations. The scalability experiments were run on randomly sampled subsets of the WTCCC HT-NBS dataset [1]. The predictive performance of greedy RLS is demonstrated on an independent test set, and the biological relevance of the results are briefly analyzed.

### Scalability experiments

In the scalability experiment, the number of training examples was held fixed at 1,000, but the number of features was incrementally increased. The considered feature set sizes were 10, 100, 1,000, 10,000, 100,000, 250,000 and 500,000. All methods implemented greedy, wrapper-based selections. The number of selected features was set to 10. By definition, greedy RLS uses LOO-CV as the selection criterion. We used the less computationally demanding 10-fold CV for the SVM-wrappers, because of the high computational costs of performing LOO for SVMs. The selection criterion for the individual features in the dataset was based on the root mean squared error (RMSE). The choice was made for computational reasons, since computing RMSE can be done in linear time, whereas computing the more commonly used AUC measure has *O*(*m*log(*m*))complexity due to a required sorting operation. RMSE as a selection criterion can be expected to work well as long as the class distributions are not very imbalanced (see e.g. [68]).

In Figure 1, we present the run-time comparisons of the two proposed variations of greedy RLS. As expected from the theory presented in the Methods section, along with the speed advantages of C++ over Python, the fast implementation turned out to be orders of magnitude faster than the space-efficient version. This performance increase comes at a cost requiring higher memory usage, hence making it infeasible to run the basic greedy RLS on the GWAS containing a very large number of training examples. For these scenarios it would be necessary to implement the space-efficient variation.

**Figure 2:**
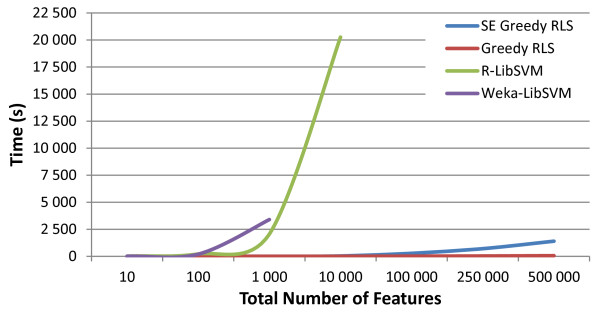
**Timing results of various wrapper-based methods.** Plot showing the comparisons between the timing results of the different feature selection implementations. Greedy RLS and space-efficient (SE) greedy RLS both used LOO, greedy feature selection and an RLS classifier, while Weka and R implemented LibSVM, used greedy forward selection as the search strategy and 10-fold CV as the selection criterion.

From the runtimes in Figure 2 it can be ascertained that other than greedy RLS, the current, commonly used algorithms for wrapper-based methods are not computationally efficient enough to scale up to entire GWAS. The R implementation of the SVM-wrapper took over 5 hours to select 10 features out of 10,000 and at 100,000features the run had to be terminated early since it exceeded the pre-determined cut-off time of 24 hours. In the commonly used Weka environment, the approach scaled worse with the program not being able to complete the selection in a 24 hour period for the dataset consisting of 10,000features. In contrast, greedy RLS computed the selection process even on 500,000 features in 1 minute while the space-efficient greedy RLS performed the feature selection process on the same dataset in under 24 minutes (see Figure 2).

### Generalization Capability

In addition to the run time comparisons, we also conducted a sample run on the entire WTCCC HT-NBS dataset to predict an individual’s risk for hypertension and to investigate whether greedy RLS can accurately discriminate between the risk classes on an independent test set. In order to reduce the variance of the results, we adopt the so-called nested CV approach (see e.g. [69-71]), in which an external CV is used for estimating the generalization capability of the learned models and an internal CV for assessing the quality of feature sets separately during each round of the external CV. First, the whole dataset was divided into three equally sized folds. Each of the three folds were used as a test set one at a time, while the remaining two were used to form a training set. Finally, the results of these three external CV rounds were averaged. The internal selection process itself with the LOO-CV criterion was run on the training sets, and up to 50 features were selected. The test folds were used only for computing the final test results for the models obtained after running the whole feature selection process.

**Figure 3:**
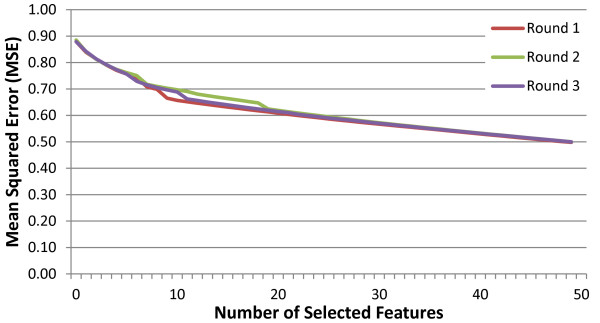
**Mean squared error for greedy RLS.** The plot displays the mean squared LOOCV errors used as a selection criteria by Greedy RLS during the three rounds of the external CV. It can be observed that as expected, the errors are consistently decreasing since the selection criterion quickly overfits to the training folds during the selection process.

In Figure 3, we present the leave-one-out cross-validated mean squared errors on the training sets in the three external CV rounds, used as the selection criterion by greedy RLS. The three selection criterion curves behave quite similarly, even if the corresponding training sets overlap with each other only by half of their size. The curves are monotonically decreasing, which is to be expected, as it is very likely that the selection criterion overfits due to the excessive number of available features to choose from (see e.g. [69] for further discussion). Clearly, they are not trustworthy in assessing the true prediction performance of the learned models. A separate test fold is thus necessary for this purpose.

**Figure 4:**
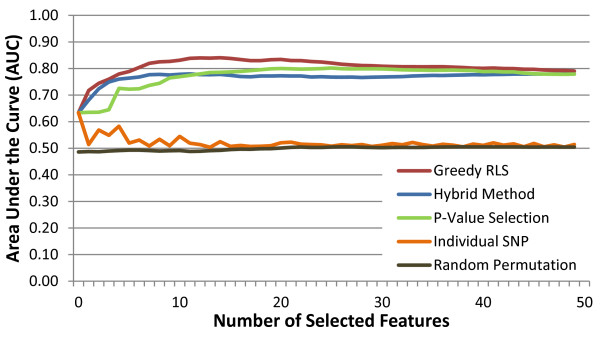
**Comparison of feature selection approaches in terms of predictive accuracy.** The prediction performances of the models learned by greedy RLS were assessed using area under the ROC curve (AUC), averaged over the three folds of an external CV. On each round of the external CV, the training set on which the features are selected consisted of 2/3, and the independent test set on which the prediction performance is measured, 1/3 of the 3,410subjects, with a stratified training/test split. The graph also displays the individual SNP AUCs for each of the variants selected by greedy RLS. Further, results are depicted for a p-value based filtering in which the top *k* most significant features were selected. We also present a curve that displays the results for the hybrid method in which greedy RLS runs on the top *k* features ranked according to their p-values. Finally, we present a random permutation on the class labels and running greedy RLS on this randomized dataset.

During each round of the external CV, after the selection process has been performed for a number of features ranging from 1 to 50, the AUCs of the learned models are evaluated on the independent test fold that was not seen during the selection (a.k.a nested CV). The results averaged over the three test folds are presented in Figure 4. At first the AUC keeps increasing with the number of selected features reaching its peak 0.84 AUC at 15 features, after which it starts decreasing. The result demonstrates that the selection process must be stopped early enough in order to avoid overfitting. Note that as observed from the Figure 3, the leave-one-out error does not provide a reliable criterion for determining the stopping point due to its use in the feature selection process. Rather, the AUC observed on an independent test fold not used during selection can be used to determine the number of features to select.

We compared the prediction performance of greedy RLS to that of two commonly used approaches in GWAS, which are both based on training a classifier on feature sets selected through filtering [3,7,18]. The reference methods start by using p-value based filtering to rank the features. The p-values were computed on the training sets using PLINK, based on Fisher’s exact test on a 3x2 contingency table of the genotypes. The filter approach is based on training a RLS classifier directly on the top *k* features having the smallest p-values. The second approach is a hybrid method, where the filters are first used to select 50 features with the smallest p-values and then greedy RLS is used to select *k* features from this set of pre-filtered features afterwards. The baseline results are based on the same three-fold CV setting as the results of greedy RLS.

As expected, the first feature selected by all of the approaches was the same since the LOO error employed by the greedy RLS as a selection criterion does not considerably differ from the statistical tests when computed for a single feature. Afterwards, however, greedy RLS begins to outperform the baseline methods, as the filter-based and hybrid approaches tend to select features that may be highly correlated with the already selected features. From Figure 4 it can be noted that while the performance of the hybrid method on the test set performs similarly to that of greedy RLS for the first couple of features, it relatively quickly begins to level off around 0.77 AUC, peaking at 0.78, below that of greedy RLS’s maximum. In contrast, the filter method requires considerably more features before its prediction performance gets close to 0.77, peaking at 0.80 before beginning to decline. The results indicate that through the use of wrapper based feature selection, it is possible to identify sets of features that have the capacity to outperform those selected by filter or hybrid methods. The total time to select the top features over the three folds of the external CV was approximately 26 minutes.

To measure the performance of the selected variants in a single-feature association analysis, the individual AUC of each of the 50 selected features was computed (see Figure 4). It can be observed that most of the single-feature AUCs are close to a random level. The maximal AUC (0.63) occurs for the first selected variant. This lack of power for the majority of the selected SNPs to distinguish between cases and controls would lead to the conclusion that the selected variants individually are not associated with the disease. On the contrary, when the combined phenotypic effect of these variants is taken into account with the RLS algorithm, much more accurate models can be trained.

To demonstrate that the experimental setup was implemented correctly, so that there is no information leak between the training and test data, we conducted a feature selection based on a random permutation of the class labels. The data and the training/test set splits were identical to those used in the original run, with the only difference being that the class labels in the dataset were randomly permuted prior to running the experiments. The top fifty features were then selected as before and the resulting AUC of the trained classifier implemented on the test set was recorded. As expected, the randomized class labels run resulted in random AUCs regardless of the number of features that were selected (see Figure 4), indicating that the results of a random labeling can not generalize beyond the original data, whereas the original SNPs have a greater ability to make accurate predictions on independent datasets.

### Feature selection results

The application of machine learning algorithms to complex GWAS datasets is not a trivial task, and there are numerous factors that can strongly alter the result in such settings. Without a solid understanding of the methodologies, it is very easy for researchers to come to incorrect conclusions about the results presented to them. Additionally, these methods can be heavily affected by any quality control procedures that are implemented. We show here that a number of the selected features are linked to prior identified factors in other published manuscripts. However, wrapper-based approaches are prone to selecting features that have unforeseen epistatic interactions amongst them and it can therefore be expected that not all of the selected features will be present in the literature. As such, while certain variants with known phenotypes [72-74], such as blood pressure, can be expected to be selected, as with any GWAS, it is likely that previously unidentified SNPs may also demonstrate disease associations.

To study the cellular mechanisms behind the selected variants, we mapped the top selected features identified by greedy RLS run on the entire cohort. To map the phenotypes we conducted a literary review of the SNPs and genes that are located within 20,000 base-pairs based on results from the dbSNP database [75]. The number of features to be analyzed, 15, was determined by the point at which the maximal AUC was obtained from the nested CV, as explained in the previous section. Of the fifteen variants, five have been identified in other publications to have either known or possible links (through gene mapping) to hypertension and related phenotypes (see Table 1): HTR3B (two variants), MIR378D1, rs10771657, SCOC.

**Table 1:** Variants selected by the greedy RLS algorithm

**SNP**	**Gene**	**Chromosome**	**Position**
rs7837736	Intergenic	8	15296703
rs1908465	Intergenic	8	15308433
rs17116117	HTR3B	11	113801591
rs10843660	Intergenic	12	30368457
rs17667894	MIR17HG	13	92014309
rs17116145	HTR3B	11	113804326
rs10771657	Intergenic	12	30359294
rs17459885	Intergenic	12	30360879
rs16837871	MIR378D1	4	5941112
rs7691494	C4orf50	4	5942649
rs6588810	ASMT	X	1753118
rs11005510	Intergenic	10	58532989
rs6840033	SCOC	4	141228861
rs10499044	Intergenic	6	107141295
rs2798360	LOC100422737	6	107148473

The list of the top 15 selected features on the entire cohort. The first column represents the SNP identifier. The second column indicates which gene the particular SNP is mapped to, or if it can not be mapped to any gene then it is marked as an intergenic sequence. The third and fourth columns are the chromosome number and base-pairs location of the SNP, respectively.

Variants with interesting mappings included MIR378D1, HTR3B, SCOC and rs10771657. MIR378D1, better known as microRNA 378d-1, is a gene located on chromosome 4 which is involved in the function of microRNA-378. It has been shown previously that mircoRNA-378 promotes angiogenesis through its over-expression and targeting of Sufu-associated pathways [76]. Angiogenesis, the process of new blood-vessels growing from existing ones, is associated with hypertension in [77]. Also, SCOC (short coiled-coil protein) has been significantly associated with hypertension [78]. HTR3B was previously identified as having a possible link to the control of blood pressure in rats, through its central influence on the sympathoinhibitory mechanism [74,79]. While this study focused on rats, it provides enough evidence to warrant HTR3B as being a candidate for examination in human-based GWAS studies. Similarly, rs10771657 was examined in other studies and identified as having a statistical association towards pulmonary function, a trait related to hypertension [80].

Nine out of the top fifteen selected features were also among the fifty features with the lowest p-values. As already discussed in previous section, the filter methods tend to select features that are correlated with each other, and therefore some of the features among the ones with the lowest p-values will not be selected by greedy RLS because of their redundancy with the previously selected features. Moreover, in contrast to the filter methods, all the features selected by greedy RLS may not be very informative individually but will be helpful for constructing a predictor when used together with other genetic features. We therefore believe that there is a strong possibility that the genetic features selected by greedy RLS are linked to the underlying biology, even if all of their disease-associations have not yet been established.

## Materials

### Study cohort

For building and testing the model we examined data from the Wellcome Trust Case-Control Consortium’s (WTCCC) study cohorts along with the set of controls from the UK National Blood Service Control Group (NBS). WTCCC is a group of 50 UK-based research groups whose aim is to better understand patterns amongst the genetic variants and their relation to disease onset [1].

From the WTCCC data cohorts we chose to examine a single case study, the Hypertension (HT) dataset in conjunction with the NBS controls set [1]. The original dataset consisted of 3,501 individuals and 500,568 SNPs distributed across 23 chromosomes that were originally sequenced with the Affymetrix 500k chip. From this set, 91 individuals and 30,956 SNPs were removed based on the exclusion lists for the associated datasets [1]. This reduced set was further filtered in PLINK based on standard quality control procedures including implementing filters that excluded features that failed the Hardy-Weinberg equilibrium in the controls with a threshold of *P*< 1×10^−3^, a minor allele frequency of 1%, a missing rate of 5%, along with a filter eliminating individuals who were missing data from more than 5% of SNPs [2,3,81-85]. After this quality control the dataset incorporated 3,410 individuals and 404,452 SNPs. As the aim of this study was to test the feasibility of the proposed algorithm, rather than the suitability of the selected features, we omitted advanced filtering methodologies such as population stratification or the adjustment of call rates to more conservative values.

### Data treatment

The RLS and SVM based methods require, that the features are encoded as numerical values. The SNP data that was used in the runs were 0, 1 and 2 corresponding to the minor allele count for the genetic feature, representing the major allele homozygote, the heterozygote and the minor allele homozygote respectively. For the scalability experiment, the runs used 1,000 examples and 10, 100, 1,000, 10,000, 100,000, 250,000 and 500,000 features. The file formats used for the data input were ARFF, binary and binary file formats for Weka, R and greedy RLS respectively.

## Conclusions

This paper is a proof-of-concept of wrapper methods being able to scale up to entire GWAS and having the capacity to perform better than the traditional filter or hybrid methods. Thorough consideration of the effects of different quality control procedures on the results, and biological validation of the found feature sets falls outside the scope of this study. The greedy RLS algorithm is the first known method that has been successfully used to perform a wrapper-based feature selection on an entire GWAS. This novel approach created a solution for an important problem, providing highly accurate results. Both the computational complexity analysis and practical scalability experiments demonstrate that the method scales well to large datasets. One critical question that remains is, what is the optimum number of features to select in such as study. While there is no definitive answer, our results indicate that even a small number of features may provide accurate prediction models.

The scalability of greedy RLS was compared to that of SVM-based wrapper methods, namely LibSVM in both the e1071 library in R and through a command line interface with the Weka software package. We demonstrate that unlike the proposed method, the other publicly available methods have too high computational runtimes to be suitable for GWAS data sets. This is not to say that there do not exist other equally valid machine learning algorithms that could handle this task. However, our work is the first known implementation of wrapper based selection that has been demonstrated to scale to entire genome scans in GWAS. Machine learning-based feature selection is a powerful tool, capable of discovering unknown relationships amongst feature subsets. However, researchers need to account for the computational complexities involved in scaling the wrapper-based feature selection methods up to GWAS. Implementation of wrapper approaches through the use of the learning algorithm as a black box inside the wrapper is simply not feasible on GWAS scale. Rather, one needs to know how to optimally implement the procedure in order to re-use computations done at different search steps and round of cross-validation. Embedding of the computations is the central key to allowing greedy RLS to scale to GWAS.

## Endnotes

^a^In the literature, the formula of the linear predictors often also contain a bias term. Here, we assume that if such a bias is used, it will be realized by using an extra constant valued feature in the data points.^b^The method is often presented in the literature as an alternative, but equivalent formulation as a constrained optimization problem.

## Competing interests

The authors declare that they have no competing interests.

## Authors’ contributions

Tapio Pahikkala and Sebastian Okser made equal contributions to this article. Tapio Pahikkala - Design, implementation, and formalization of the greedy RLS algorithm and drafting of the manuscript. Sebastian Okser - Design of the experiments, implementation of the algorithm on the GWAS, analysis of the results and drafting of the manuscript. Antti Airola - Participated in the development process of greedy RLS, experimental design and implementation, and drafting of the manuscript. Tapio Salakoski - Supervision of the research and methodology design. Tero Aittokallio - Conceived the study, participated in the design of the experiment and in drafting of the manuscript. All authors read and approved the final manuscript.
